# Leukemia inhibitory factor (LIF) withdrawal activates mTOR signaling pathway in mouse embryonic stem cells through the MEK/ERK/TSC2 pathway

**DOI:** 10.1038/cddis.2015.387

**Published:** 2016-01-14

**Authors:** M Y Cherepkova, G S Sineva, V A Pospelov

**Affiliations:** 1 Laboratory of Molecular Mechanisms Cell Differentiation, Institute of Cytology, Russian Academy of Sciences, St. Petersburg, Russia

## Abstract

Leukemia inhibitory factor (LIF) is indispensable to maintain the pluripotent state of mouse embryonic stem cells (ESCs), but the mechanisms underlying the role of LIF/STAT3 pathway are yet poorly understood. Here we first showed that the LIF/STAT3-regulated signaling pathway contributes to the maintenance of self-renewal and pluripotency of mouse ESCs by suppressing mTOR (mammalian target of rapamycin), which is necessary for early differentiation. When LIF is withdrawn from culture medium, the mTOR activity rapidly increases as detected by phosphorylation of its targets – ribosomal protein S6 and translation factor 4EBP1. In turn, suppression of STAT3 phosphorylation on Tyr-705 by a specific small molecule WP1066 also activates phosphorylation of the mTOR target S6 ribosomal protein. LIF removal strongly activates ERK activity indicating that ERK can be involved in either direct phosphorylation of mTOR or phosphorylation of an upstream negative regulator of mTOR – TSC1/TSC2 proteins. According to western blotting data, LIF withdrawal leads to phosphorylation of TSC2 protein thereby relieving its negative effect on mTOR activity. mTOR activation is accompanied by a decrease of pluripotent gene expression *Oct-4, Nanog, Sox2* and by an augmentation of *fgf5* gene expression – a marker of post-implantation epiblast. Together, these data indicate that LIF-depleted mouse ESCs undergo a transition from the LIF/STAT3-supported pluripotent state to the FGFR/ERK-committed primed-like state with expression of early differentiation markers mediated through activation of mTOR signaling.

Embryonic stem cells (ESCs) are pluripotent cells derived from the early blastocyst that are capable of self-renewing for a long time *in vitro*. The mechanisms underlying pluripotency and self-renewal are of great interest, and a number of signaling pathways (LIF/STAT3, Ras/MAPK, BMP/Smad, PI3K/Akt and Wnt/*β*-cathenin) have been shown to contribute to the circuitry of ESCs state.^1, 2, 3^ LIF/STAT3 (Leukemia Inhibitory Factor/Signal Transducer and Activator of Transcription-3) signaling pathway is one of the first described pathways regulating pluripotency in mouse ESCs.^4^ LIF is a cytokine from the IL-6 family, which acts through the LIFR/gp130 receptor and activates STAT3 transcription factor at Tyr-705 that provides activation of pluripotency genes expression and downregulation of such differentiation markers as *Gata3*, *Gata4*, *T/brachyury* and *Eomes*.^5^ It has been also shown that LIF can activate Ras-MAPK and PI3K-Akt through the involvement of gp130 adaptor protein.^6, 7^ Thus LIF/STAT3 signaling pathway promotes self-renewal and blocks the differentiation of mouse ESCs. Respectively, LIF removal from culture media is the first step to change mouse ESCs pluripotent state and to direct to differentiation. It is known that suppression of MEK/ERK by pharmacological inhibitors promotes their self-renewal, whereas ERK activation is essential for differentiation induction in embryonic stem cells suggesting a role of LIF pathway in maintaining the low levels of the ERK activity in undifferentiated cells.^8^

mTOR (mammalian target of rapamycin) signaling pathway has an important role in a variety of cell processes including cell growth, translation regulation, cell metabolism, cell cycle regulation and autophagy. This signaling pathway integrates signals from growth factor receptors (insulin and IGF), energy status, amino acids abundance and several stress factors. The key protein of this pathway mTOR kinase was found to be a key component of two different molecular complexes: mTORC1 (rapamycin sensitive complex) and mTORC2 (rapamycin-insensitive complex).^9^ mTOR is essential for growth and proliferation in early mouse embryo and proliferation of mouse ES cells. Thus, disruption of *mTOR* gene results in early post-implantation lethality; moreover, mES cells fail to be obtained from *mTOR*^*−/−*^ blastocysts.^10, 11^ Normal function of mTOR-signaling pathway is also essential for trophoblast development. There are contradictory data on the role of mTOR in human ESCs: according to Zhou *et al.* mTOR supports long-term self-renewal, while other reports suggest that mTOR-mediated activation of p70-S6K induces differentiation.^12, 13^ DEPTOR, a negative regulator of mTOR signaling, has an important role in maintenance of pluripotent state of embryonic stem cells and its level dramatically decreases with differentiation of mouse ESCs.^14^

Here, we examined the activity of mTOR signaling pathway under conditions that permit self-renewal of mESCs (in the presence of LIF), and under conditions that promote differentiation (in the absence of LIF). mTOR activity in the mTORC1 and mTORC2 complexes increases after LIF withdrawal from the medium indicating that LIF/STAT3 signaling negatively effects the activity of mTOR. To understand the mechanism of mTOR activation, we checked the phosphorylation state of TSC2, an upstream negative regulator of mTOR. It turned out that in LIF-depleted cells the activated ERK is involved in phosphorylation of TSC2 protein thereby relieving its negative effect on mTOR activity. qRT-PCR analysis showed that the activation of mTOR upon LIF withdrawal occurs simultaneously in parallel with a decrease in transcription of genes *oct4, nanog, klf4* and *sox2* and by an increase of *fgf5* gene expression – the marker of post-implantation epiblast and primed pluripotent state. Treatment with MEK1,2 inhibitor PD0325901 canceled the mTOR activation thereby implying the involvement of MEK-ERK pathway in mTOR activation. Together, these data indicate that LIF depletion of mouse ESCs induces a transition from LIF/STAT3-supported pluripotency to FGFR/ERK-committed primed-like state mediated through activation of mTOR signaling.

## Results

### LIF withdrawal has little effect on the cell cycle parameters and viability of mESCs, but suppresses the expression of pluripotency genes

**Figure 1:**
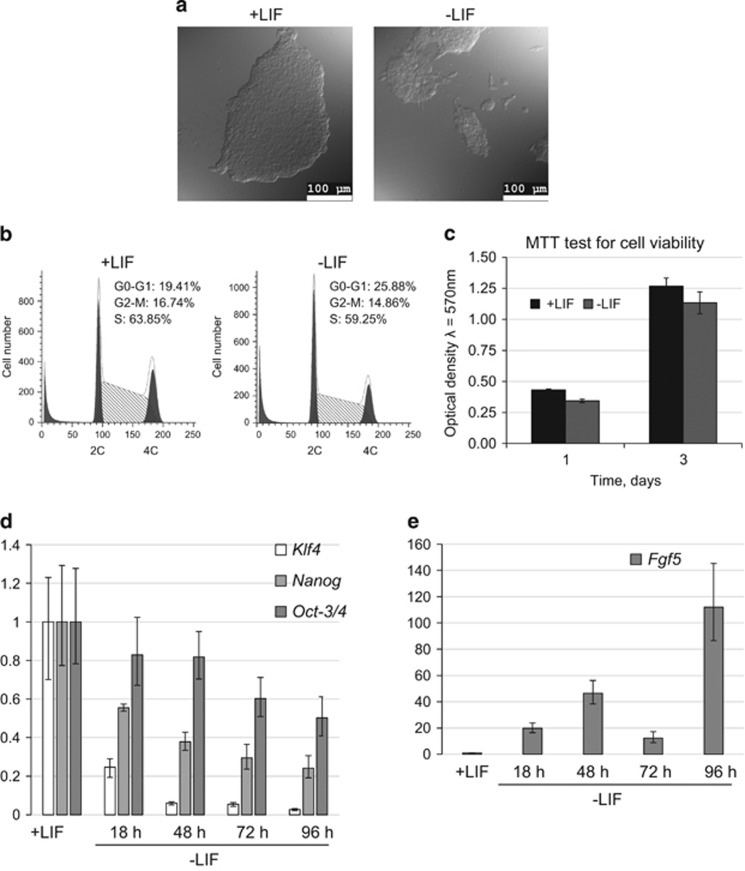
LIF withdrawal has little effect on the cell cycle parameters and viability of mESCs but suppresses the expression of pluripotency genes. (**a**) Morphology of mESCs grown in the presence and absence of LIF for 24 h. DIC microscopy, scale bar, 100 *μ*m. (**b**) Flow cytometry analysis of cell cycle parameters of mESCs grown in the presence of LIF and LIF-depleted for 24 h. (**c**) MTT-test for cell viability of mESCs grown as in (**b**). The experiment was repeated at least three times and the one is shown with the mean±S.D. for six replicates is given. (**d**) mRNA expression level of *Klf4, Nanog* and *Oct4* genes in undifferentiated mESCs (+LIF) and in LIF-depleted for 18, 48, 72 and 96 h mESCs. Data were obtained by qRT-PCR, the expression was normalized to *Gapdh* gene expression level in each sample. The experiment was repeated three times, the results of one experiment are given with the error bars correspond to the S.E.M. calculated for three replicates. (**e**) Expression levels of *fgf5* gene mRNA. Experimental details are the same as in (**d**)

To maintain the self-renewal and pluripotency, mESCs need to keep a balance of the activity of different signaling pathways. Of them, an important role belongs to a signaling pathway regulated through LIF/STAT3. It has been shown that LIF is necessary to ensure prolonged proliferation of mESCs, while retaining their pluripotent properties.^4^ First, we compared the morphological and cell growth characteristics of mESCs growing in the absence of LIF. According to this data, within 24 h after LIF withdrawal the morphology of mESC E14TV2 cell line acquires some features characteristic for differentiating cells. Undifferentiated mESCs grow in compact and dense colonies, which are becoming less dense and more flattened after LIF withdrawal with the increasing number of individual separately growing cells (Figure 1a). Nevertheless, removal of LIF did not lead to significant changes in parameters of mESCs cell cycle. Flow cytometry data show that in the absence of LIF there was a 5% increase in the number of G_0_–G_1_ phase-engaged cells and ~4% decrease of S-phase cells (Figure 1b). Also, lack of LIF had only slight effect on the viability of mESCs as assessed by MTT-test (Figure 1c).

In contrast to the viability and cell cycle parameters, qRT-PCR analysis demonstrates that the LIF withdrawal leads to a decrease in the expression levels of pluripotency gene markers. Within 18 h after removal of LIF, a reduction of mRNA transcripts of *Klf-4* gene does occur followed by a decrease of *Oct-3/4* and *Nanog* gene expression at 48 h (Figure 1d). Concomitantly with downregulation of *Klf4, Oct-3/4* and *Nanog* expression, a marker of epiblast differentiation, *Fgf5* gene, a direct target for Klf4 repression,^15^ gradually upregulates (Figure 1e). The observed changes in the profile of gene expression evidence for an onset of differentiation program in E14TV2 mESCs in response to LIF withdrawal and for the acquisition of more primed-like phenotype by these cells. Of interest, among the checked genes *Klf4* is the first responding gene upon LIF removal, likely because its promoter is a direct target for LIF-regulated transcription factor STAT3. Therefore, one can suggest that in LIF-depleted mES cells initiation of differentiation and early changes in the pattern of gene expression proceeds through Klf4-mediated modulation of a network of transcription factors having a role in pluripotency.^16^

Thus, although an onset of differentiation triggered by LIF removal downregulates the expression of a set of pluripotent genes, these events do not significantly affect the viability and cell cycle parameters of mESCs.

### LIF withdrawal results in the activation of mTOR-signaling pathway

LIF, a member of IL-6 cytokine family, is required to maintain the undifferentiated state of the cultured mouse ES cells. Available data indicate that LIF inhibits commitment of mESCs to differentiation and development of embryoid bodies into visceral and parietal endodermal cells, while LIF withdrawal attenuates pluripotency gene expression and induces expression of some differentiation markers.^17^ Our experiments support these data and show that LIF withdrawal is followed by the decrease of mRNA levels of *Nanog, Klf4,* and *Oct-3/4* genes and induction of *Fgf5* gene expression, which is known to be an early epiblast marker gene (Figures 1d and e).

**Figure 2:**
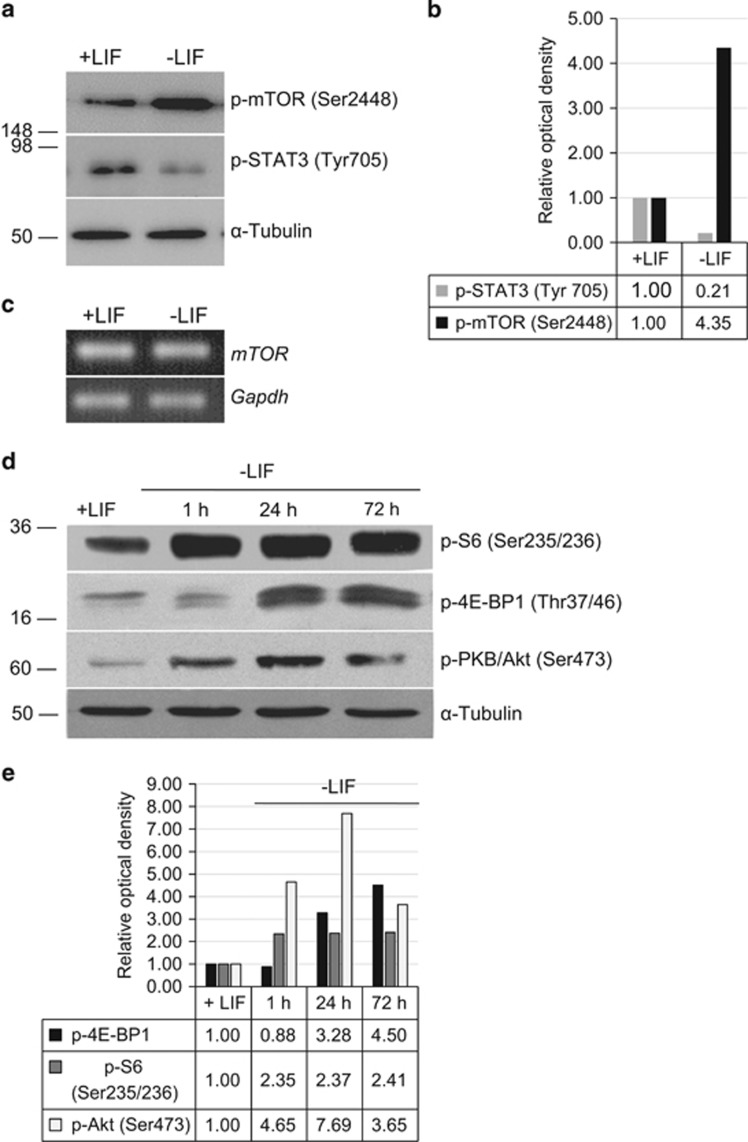
LIF withdrawal leads to activation of mTOR-signaling pathway. (**a**) Western blot of mTOR autophosphorylation at Ser-2448 and a decrease of STAT3 phosphorylation at Tyr-705 as determined 24 h after LIF withdrawal. *α*-Tubulin was used as loading control. The representative of experiments repeated at least three times is shown. (**b**) Densitometry of bands intensity from (**a**). (**c**) *Mtor* gene mRNA level does not change in mESCs grown without LIF for 24 h. *Gapgh* mRNA level was used as loading control. (**d**) Western blot analysis of mTOR-target proteins extracted from mESCs grown in the presence of LIF (+LIF) or without of LIF for 1, 24 and 72 h (-LIF) with antibodies to p-S6 (Ser235/236), p-4E-BP1 (Thr37/46), p-PKB/Akt (Ser473). *α*-Tubulin was used as loading control. The representative of experiments repeated at least three times is shown. (**e**) Densitometry of bands intensity from (**d**)

**Figure 3:**
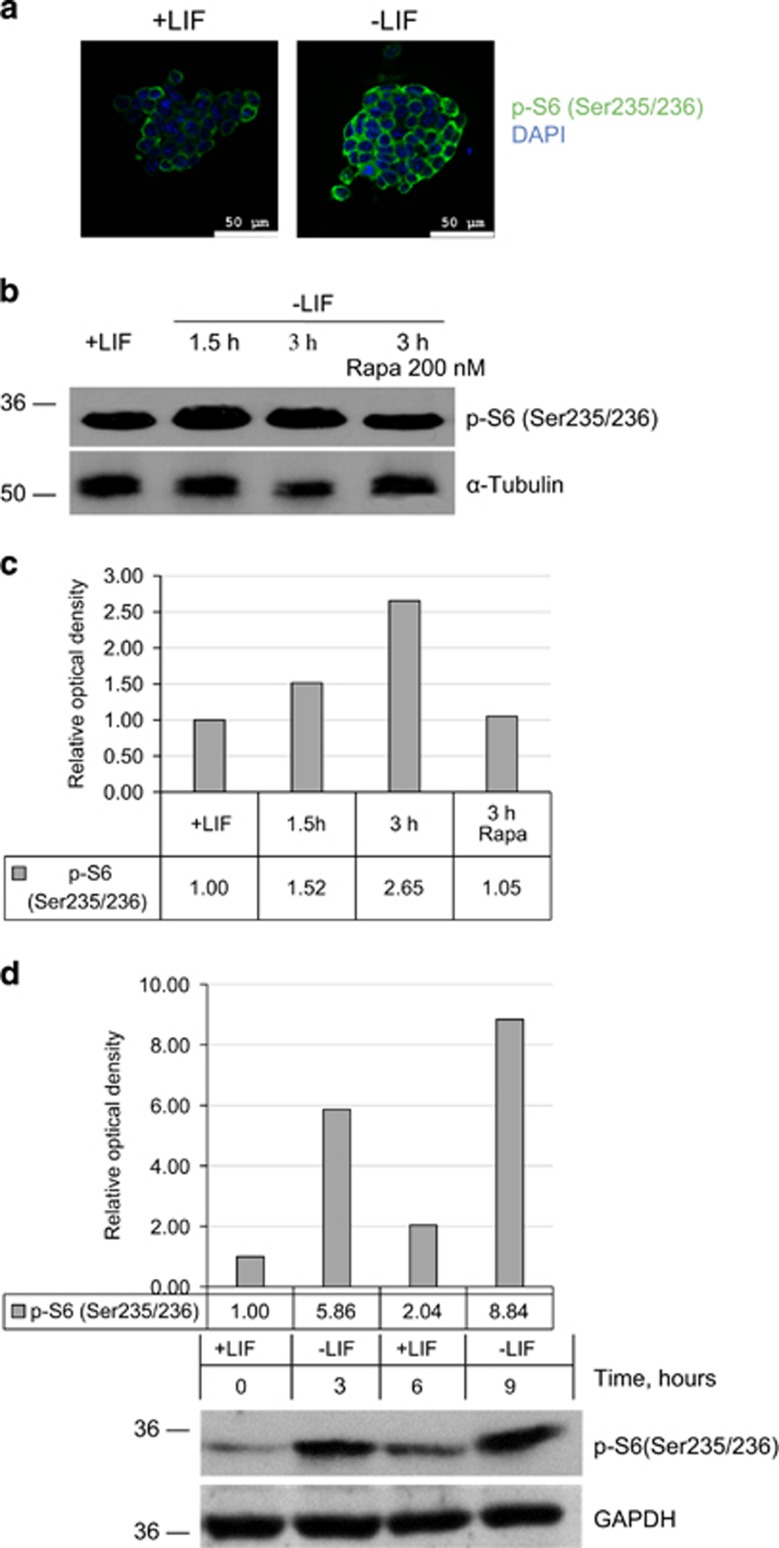
Phosphorylation of S6 protein in LIF-depleted mESCs is reversible and decreases upon re-addition of LIF. (**a**) Phosphorylated p-S6 protein (Ser-235/236) accumulates in mESCs after LIF withdrawal for 24 h. Immunofluorescent staining with antibodies against p-S6 (Ser-235/236) – green, DNA staining with DAPI – blue. Scale bar, 50 *μ*m. (**b**) mTORC1 inhibitor rapamycin (200 nM, 3 h) suppresses phosphorylation of mTOR-target S6 protein (Ser-235/236) caused by LIF withdrawal. The representative of experiments repeated at least three times is shown. (**c**) Densitometry of bands intensity from (**b**). (**d**) S6 protein phosphorylation at Ser-235/236 is reversible and decreases after LIF re-addition for 3 h. GAPDH was used as loading control (bottom). Densitometry of bands intensity is given in (**d**) (upper). The representative of experiments repeated at least three times is shown

mTOR signaling pathway is known to have an important role in a variety of cell processes including cell growth and proliferation, translation regulation and cell metabolism, cell cycle regulation and autophagy. To find out whether the mTOR signaling is involved in the initiation of differentiation induced by LIF withdrawal, we compared the levels of mTOR-kinase activity in LIF-supplemented and LIF-depleted cells. According to obtained results a significant phosphorylation of mTOR kinase at Ser2448 is observed upon LIF withdrawal, although transcription of *mTOR* gene does not change (Figures 2a–c). To ensure that mTOR phosphorylation leads to its activation, we checked phosphorylation state of mTORC1 and mTORC2 target proteins. The increase of mTOR kinase activity was confirmed by accumulation of phosphorylated mTORC1 target proteins – eukaryotic translation initiation factor 4E-Binding Protein 1 (4E-BP1) at Thr37/46 and S6 ribosomal protein at Ser235/236, as well as a mTORC2 target – PKB/Akt kinase phosphorylated at Ser473 (Figures 2d and e). Phosphorylated pS6 protein accumulates in the cytoplasm of LIF-depleted mouse ESCs as evidenced by immunofluorescence data (Figure 3a). These results unambiguously indicate that LIF withdrawal is capable of inducing the activation of both mTORC1 and mTORC2 complexes (Figures 2 and 3).

**Figure 4:**
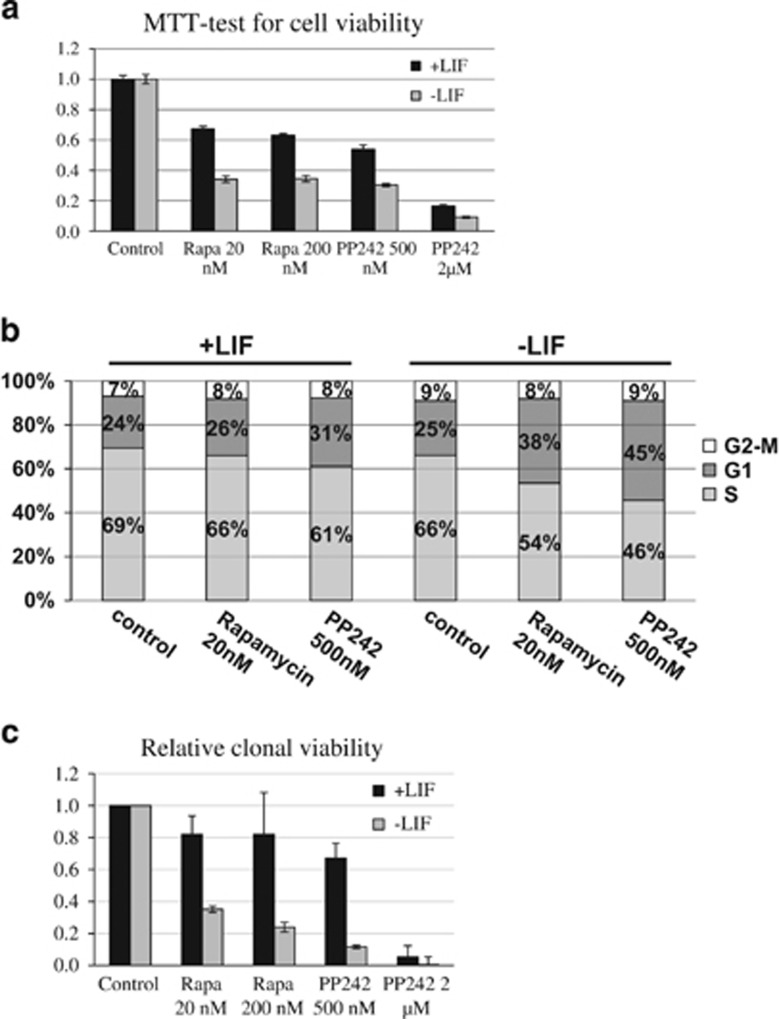
mTOR inhibitors affect to a greater extent the viability, cell cycle parameters and cloning efficiency of LIF-depleted mESCs. (**a**) MTT-test for cell viability of mESCs grown in +LIF or −LIF medium and treated with Rapamycin (Rapa) or PP242 for 24 h. The experiment was repeated at least three times and the one is shown with the mean±S.D. for six replicates is given. (**b**) Cell cycle parameters of mESCs grown in +LIF or -LIF medium and treated with Rapamycin (Rapa) or PP242 for 24 h. (**c**) Clonal growth of mESCs grown in +LIF or −LIF medium and treated with Rapamycin (Rapa) or PP242 for 24 h. The experiment was repeated at least three times and the one is shown with the mean±S.D. for three replicates is given

To confirm that phosphorylation of pS6 is mTORC1-dependent event, LIF-depleted cells were treated with an allosteric mTORC1-specific inhibitor rapamycin. Indeed, pS6 phosphorylation proved to decline upon rapamycin treatment (Figures 3b and c). Moreover, rapamycin partially restores the expression level of *Oct4* and *Nanog* genes in LIF-depleted mESCs indicating the involvement of mTORC1 in regulation of these genes (Supplementary Figure 1). In addition, phosphorylation of S6 ribosomal protein decreased after re-addition of LIF, thus proving the activation of mTOR pathway is reversible (Figure 3d). Noteworthy, phosphorylation of mTOR target proteins have different time schedule: PKB/Akt at Ser473 and S6 at Ser235/236 are phosphorylated as soon as 1 h after LIF withdrawal, while 4E-BP1 phosphorylation increases only after 24 h (Figures 4a and b).

Interestingly, suppression of STAT3 activity, as assessed by decreased Tyr-705 phosphorylation in LIF-supplemented mESCs by a specific small molecule WP1066, also activates phosphorylation of mTOR target S6 ribosomal protein implying that STAT3 is an upstream negative factor for S6 phosphorylation (data not shown). This confirms a conclusion that mTOR is under negative control of LIF/STAT3 pathway.

### Effect of mTORC1 inhibitor rapamycin on LIF-supplemented and LIF-depleted mouse ESCs

**Figure 5:**
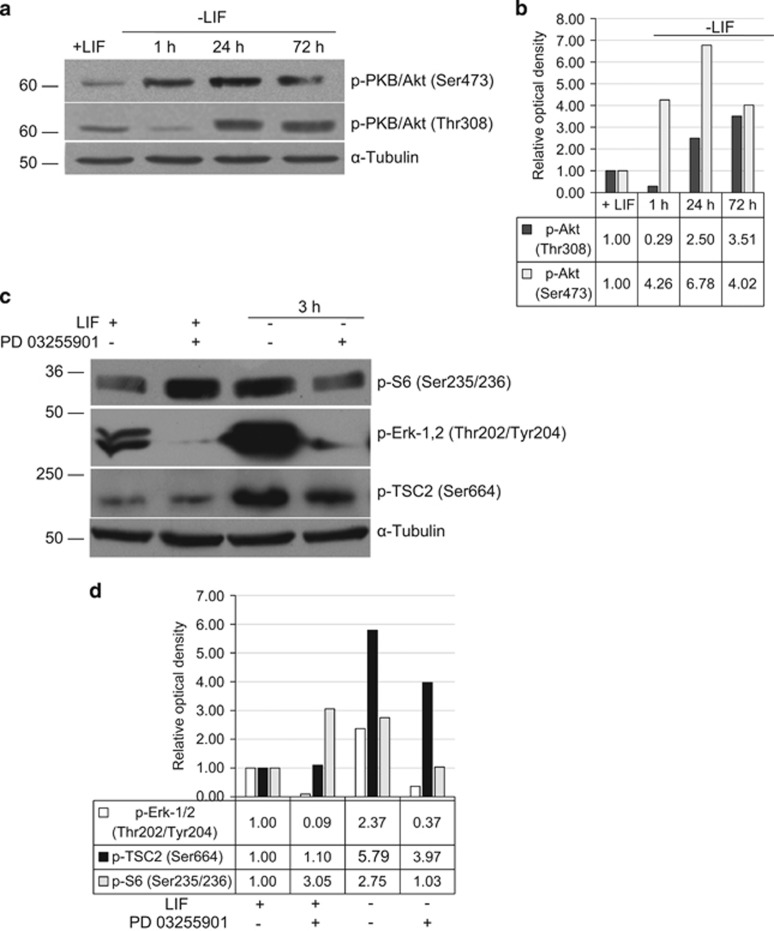
MEK-ERK1/2 activation is necessary for mTOR phosphorylation induced by LIF withdrawal. (**a**) PKB/Akt is phophorylated at Ser473 but not at Thr308 in LIF-starved for 1 h mESCs. Twenty-four hours after LIF withdrawal both sites of PKB/Akt are phosphorylated. The representative of experiments repeated at least three times is shown. (**b**) Densitometry of bands intensity from (**a**). (**c**) LIF withdrawal for 3 h leads to ERK1/2 phosphorylation (Thr202/Tyr204) and to inhibitory phosphorylation of TSC2 Thr664 – a negative mTOR regulator. Treatment with MEK1/2 inhibitor PD0325901 for 3 h suppresses both pS6 and TSC2 phosphorylation. The representative of experiments repeated at least three times is shown. (**d**) Densitometry of bands intensity from (**c**). *α*-Tubulin was used as loading control

An increase of mTOR kinase activity induced by LIF withdrawal accompanies an onset of mESCs differentiation. These events appear to be tightly linked and evidence for a functional significance of mTOR-pathway in triggering differentiation. To assess the role of mTOR-signaling pathway in mES cells, we used two inhibitors with different mechanisms and specificity: rapamycin (allosteric inhibitor of mTORC1) and PP242 (ATP-competitive inhibitor of mTOR kinase, which suppresses both mTORC1 and mTORC2 complexes). In accordance with the increasing role of mTOR after LIF withdrawal, the LIF-deprived mESCs become more sensitive to mTOR inhibition. Rapamycin and PP242 suppress the clonal growth and cell viability of LIF-depleted mESCs in more than twice as efficient as compared with LIF-supplemented cells (clonability and MTT assays) (Figures 5a and c). Similar results have been received by using flow cytometry for determining the cell cycle parameters of mTOR inhibitor-treated cells (Figure 5b). Both rapamycin and PP242 inhibitors have little effect on the ratio of G1, S and G2 phases in LIF-supplemented cells, while LIF-deprived cells respond to this treatment by a significant accumulation of G1-phase cells and a decrease of S-phase cells (Figure 5b). Together, these results can be interpreted to mean that mTOR activity is more essential for the viability and proliferation of mESCs, which have changed their pluripotent state after LIF withdrawal (−LIF), than for +LIF cells.

### MEK-ERK activity contributes to mTOR activation in LIF-depleted mouse ESCs

Available data evidence that activity of ERK1/2 kinases is not required for self-renewal of mESCs cultured in the presence of LIF. Moreover, it has been recently shown that a combination of MEK/ERK and GSK3*β* inhibitors is sufficient to maintain self-renewing properties of mouse ESCs (2i protocol).^18^ To elucidate a role of ERK1/2 in upregulation of mTOR signaling, we checked the phosphorylation state of the ERK1/2 on Thr202/Tyr204 residues in LIF-depleted mESCs. Data presented in Figures 4c and d show that undifferentiated +LIF cells have a moderate level of the ERK phosphorylation. When removing LIF from the cell culture medium, ERK1/2 become greatly phosphorylated and is capable of phosphorylating its targets, including those involved in mTOR regulation. One of the key upstream proteins regulating mTOR is Tuberin, a product of the *TSC2* tumor suppressor gene. Tuberin is an important regulator of cell proliferation and tumor development as well as a negative regulator of mTOR.^19^ Tuberin can be directly phosphorylated at Thr1462 by PKB/Akt that relieves its inhibitory effect on mTOR mediated through Rheb. Tuberin also can be inhibitory phosphorylated at Ser664 by ERK1,2 that leads to abrogation of mTOR inhibition. Undifferentiated cells have low levels of TSC2 phosphorylation that correlates with a low level of mTOR activity (Figures 4c and d). Very strong activation of ERK in LIF-depleted mESCs promotes phosphorylation of TSC2 and, although MEK/ERK inhibitor PD0325901 does not reduce the pTSC2 content to zero level, nevertheless that reduction is sufficient to decrease the phosphorylation of pS6, a target of mTORC1. Together, these data indicate that LIF-triggered signaling may execute a negative control on the activity of mTOR by acting through the MEK/ERK/TSC2 pathway. In the absence of LIF mTOR is activated and this provides conditions for cell growth and differentiation, while simultaneously reducing the level of pluripotent gene expression *Oct-4*, *Nanog* and *Sox2*.

### LIF-depleted mouse ES cells accumulates LC3 but the process of autophagy is likely interrupted at the stage of phagosome/lysosome fusion

**Figure 6:**
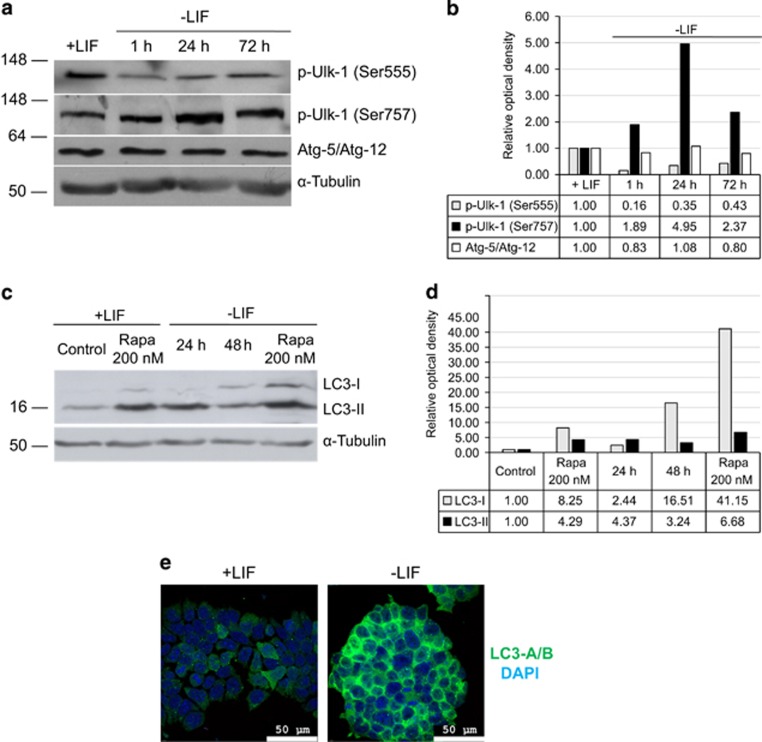
mTOR activation after LIF withdrawal does not lead to inhibition of LC3 expression. (**a**) Western blot analysis of Ulk-1 phosphorylation at Ser555 and Ser557. The Atg-5/Atg-12 heterodimers are detected in mESCS regardless of LIF presence. *α*-Tubulin was used as loading control. The representative of experiments repeated at least three times is shown. (**b**) Densitometry of band intensity in **a**. (**c**) Western blot analysis of LC3-I and LC3-II forms in mESCs cultured in the presence of LIF or without LIF. Treatment by rapamycin was during 24 h. *α*-Tubulin was used as loading control. The representative of experiments repeated at least three times is shown. (**d**) Densitometry of band intensity in **c**. (**e**) Intracellular distribution of LC3 protein. Immunofluorescent staining with antibodies against LC3 protein (green) and DNA staining with DAPI (blue)

There are many data evidencing that mTOR signaling pathway is one of the key antagonistic regulators of macroautophagy.^20^ In most cases under favorable nutritional conditions, when mTOR is active, the level of autophagy is very low, while the lack of nutrients or inhibition of mTORC1 activity by rapamycin induces autophagy.^11, 21^ Recent data show that autophagy can be fruitful for maintenance of stemness in mesenchymal and hematopoietic stem cells, particularly for removing the excess of unused or damaged proteins. We were interested to know how activation of mTOR signaling upon LIF withdrawal can affect the autophagy by assessing the expression of a key autophagy marker LC3 (Atg8). Firstly, we checked the phosphorylation state of Ulk-1 (Atg-1), which is a primary target for positive (pAMPK) or negative (mTOR) regulation of autophagy.^22^ Although Ulk-1 has many sites for its phosphorylation, the most important site for AMPK-dependent activation is Ser555, whereas phosphorylation on Ser757 by mTOR kinase leads to a suppression of autophagy. As expected, LIF withdrawal stimulates Ulk-1 phosphorylation on Ser757 that reflects upregulation of mTOR activity (Figures 6a and b). In turn, phosphorylation of Ulk-1 on Ser555 decreases in these conditions. Thus, the observed pattern of Ulk-1 phosphorylation would seem to correspond to the low level of autophagy. To prove this point, we examined the content of LC3 protein (Atg8), which is a component of autophagosomes, and its intracellular distribution in the absence of LIF. According to western blot (Figures 6c and d) and immunofluorescent data (Figure 6e), LC3 accumulates in the cytoplasm of -LIF cells. There is also the conversion of LC3-I (cytosolic form) to LC3-II (membrane-bound lipidated form). However, immunofluorescence analysis for the co-localization of LC3 and a lysosomal marker LAMP1 as an evidence of autophagy progression failed to give a positive result (data not shown). This may mean that LIF-depleted cells are in a state of arrested autophagy, when LC3 accumulates in autophagosomes but their fusion with lysosomes does not occur. In addition, western blot analysis performed to examine the content of Atg5 and Atg12 heterodimers in LIF-depleted cells shows almost the same amount of the heterodimers in LIF-supplemented and LIF-depleted mouse ESCs (Figures 6a and b).

## Discussion

Binding of LIF to the LIFR/gp130 complex can trigger at least three signaling pathways: Janus kinase JAK/STAT3; phosphoinositide 3-kinase (PI3K)/AKT; and mitogen-activated protein kinase (MAPK).^23^ The parallel LIF signaling through JAK/STAT3 and PI3K pathways maintains the pluripotent state, whereas ERK signaling appears to destabilize it.^4^ Correspondingly, the level of MEK/ERK activity is critical for maintenance of pluripotency, since FGF stimulation of the ERK1/2 signaling cascade triggers transition of pluripotent embryonic stem cells from self-renewal to lineage commitment, with Fgf4 being the major stimulus activating ERK in mouse ES cells.^8^ This explains why MEK/ERK inhibitors help to support undifferentiated state and pluripotency of mESCs.^18^

To induce differentiation, mouse ESCs are usually transferred to the LIF-free medium supplemented with a set of growth factors that promote differentiation to a certain lineage. LIF removal is one of the first triggers to commit differentiation, albeit it has a slight effect on the viability and proliferation characteristics (Figure 1). However, LIF withdrawal for 24 h lengthens the G1 phase as reported earlier.^24, 25^ Overall, this approach offers the unique opportunity to study switching over the signaling pathways controlling self-renewal, pluripotency and differentiation. Thus, despite a weak effect on the cell cycle parameters the expression of genes determining pluripotent state drastically decreases, and the change of mESC morphology takes place as well (Figure 1). This may mean that the expression of genes *Oct4, Klf4* and *Nanog* is not directly involved in proliferation of mESCs and is much more important for maintenance of the pluripotent state. Interestingly, the inducible activation of fibroblast growth factor receptor 2 (FGFR2) is sufficient to trigger *Nanog* gene downregulation through activation of the MEK/ERK pathway and differentiation of mESCs to primitive endoderm.^26^ However, this FGF2-receptor activation has minimal effect on other pluripotency genes, including *Oct4* and *Sox2*.

We showed here that LIF withdrawal rapidly activates mTOR activity implying that LIF/STAT3 signaling is involved in suppression of the mTOR pathway (Figure 2). The mTOR suppression can be carried out via PI3K/PKB/Akt and/or through MEK/ERK pathways. So, we checked phosphorylation of PKB/Akt and ERK in LIF-free medium conditions (Figures 4a and b). As for PKB/Akt, its phosphorylation at Thr-308 (PI3K/PDK1-dependent site) does not occur during the first 1 h, while phosphorylation at Ser-473 (mTORC2-dependent site) increases rapidly upon LIF withdrawal (1 h). Thus, the PKB/Akt does not participate in mTOR activation at least for 1 h after LIF withdrawal. Since the PKB/Akt itself is first phosphorylated by PDK1 kinase on Thr308, and then by mTORC2 complex on Ser473, it is unlikely that the phosphorylation of mTOR on Ser2448 is due to PKB/Akt. To this end, mTOR phosphorylated on Ser2448 is predominantly a part of the mTORC1 complex, whereas mTORC2 complex contains Ser2481-phosphorylated mTOR kinase.^27^ As to the contribution of ERK in mTOR acivation, the ERK phosphorylation rapidly and greatly increases after LIF withdrawal, suggesting that the activation of FGFR/MEK/ERK pathway may contribute to mTOR phosphorylation. Indeed, very strong activation of ERK in LIF-depleted mESCs correlates with TSC2 phosphorylation and a MEK/ERK inhibitor abolishes it (Figures 4c and d). Thus, LIF-triggered signaling may limit the activity of mTOR due to maintaining a low level of ERK activity in undifferentiated mESCs, and upon LIF withdrawal quick reactivation of MEK/ERK/TSC2 pathway takes place.

Available data show that high mTOR activity decreases the autophagic flux, therefore, of interest to know how LIF withdrawal induced mTOR activation affects the level of autophagy in mESCs. There are recent data focusing on the role of autophagy in cell reprogramming. In particular, there is a robust induction of autophagy during the reprogramming of mouse fibroblasts to induced pluripotent stem cells (iPSCs) by four transcription factors (Sox2, Oct4, Klf4 and c-Myc). This process occurs independently of p53 activation, and is mediated by the synergistic downregulation of mTORC1 and the induction of autophagy-related genes.^28^ Another work on this issue shows that *mTOR* is downregulated by Sox2 at an early stage of iPSC generation and that this transient downregulation of *mTOR* is required for the reprogramming took place. Herewith, the Sox2 binds to a repressive region on the *mTOR* promoter and recruits the NuRD complex to mediate transcriptional repression.^29^ Thus, activation of autophagy is the productive event in a transition from somatic to pluripotent (iPSC) state. In case of the exit from the pluripotent state induced by LIF withdrawal and described in the present work, the mTOR activation does not suppress a marker of autophagy LC3 or its conversion to membrane-bound LC3-II form (Figures 6c and d). But our attempts to co-localize LC3 and a lysosomal marker LAMP1 were unsuccessful. We suggest that LIF-depleted cells appear to exist in a state of arrested autophagy when LC3 can accumulate in autophagosomes but their fusion with lysosomes does not occur as was recently shown for human MSC cells.^30^ In addition, accumulation of LC3 and its membrane-bound form LC3-II takes place in case of treatment of human ESC by bafilomycin A1 (Cho *et al.*^31^), which blocks autophagolysosome formation.^32^

The interpretations of the observed phenomena can be as follows. Upon LIF withdrawal, mESCs are likely to undergo a transition from naive pluripotent state to so-called primed pluripotent state characteristic for rodent EpiSCs and primate ESC cells.^33^ LIF-depleted cells, similar to primed pluripotent cells, still express, albeit on a low level, the canonical pluripotency genes *Oct-4, Sox2* and *Nanog*, but not *Klf4*, and also can express specification markers *Fgf5* and *T/brachyury* (Figure 1). The EpiSCs can be reprogrammed to naive pluripotency by transfection with just a single factor, Klf4.^34^ Correspondingly, LIF maintains pluripotency in mouse ESCs, but not in human ESCs, thereby reflecting a distinct (naive and primed, respectively) origin of these stem cells.^35^ Interestingly, mTOR supports long-term self-renewal of human ESCs that are analogous to rodent EpiSCs and correspond to primed pluripotent state.^12, 36, 37, 38^

LIF withdrawal downregulates the expression of STAT3/Klf4 target pluripotent genes *Oct-4, Nanog, and Sox2*. As Sox2 negatively regulates the *mTOR* gene promoter activity,^29, 39^ the shutdown of Sox2 can upregulate *mTOR* transcription, although this mechanism cannot explain the immediate mTOR activation observed after LIF withdrawal. Rather, the interplay of LIF/STAT3 and FGFR/ERK pathways determines mTOR activity level, as well as the outcome of the pluripotent cell state: naive or primed pluripotency. In naive ESCs, LIF/STAT3 pathway is dominant over FGFR/ERK,^40^ but with LIF withdrawal the FGFR/ERK pathway is activated, leading to phophorylation of a negative mTOR kinase regulator, Tuberin (TSC2), and eventually to activation of mTOR.

In summary, LIF withdrawal causes a reversible transition of mESCs from naive pluripotent state to FGFR/ERK-committed early differentiation mediated through activation of mTOR signaling. The resulting state has some features characteristic for the primed pluripotency.

## Materials and Methods

### Cell culture

Mouse embryonic stem E14TV2 cell line was maintained on tissue culture dishes (Corning, Amsterdam, The Netherlands) coated with 0.2% porcine gelatin (Sigma, Munchen, Germany) in a Dulbecco's modified Eagle's medium DMEM/F12 (1 : 1) (Gibco, Moscow, Russia) supplemented with 0.1 mM 2-mercaptoethanol (Sigma), 10% fetal bovine serum (HyClone, Biolot, St. Petersburg, Russia), and 1000 units/ml of recombinant murine LIF at 37 °C in atmosphere of 5% CO_2_. Recombinant mLIF was expressed using an expression vector pGEX-2Т-hLIF in *Escherichia coli* and was further purified on a Glutathione Sepharose column. For clonal viability assay cells were seeded in a clonal density (500 cell per well on a six-well plate) and were Giemsa-stained and calculated after 6 days of culture in different media. The mTOR inhibitors rapamycin and pp242 were purchased in Calbiochem (Millipore, Darmstadt, Germany).

### FACS analysis of cell cycle distribution

For cytometric analysis of cell cycle distribution, cells were harvested, washed with phosphate-buffered saline (PBS) and incubated for 30 min in PBS containing 0.01% of saponin (Sigma) at room temperature. Cells were washed twice with PBS and incubated with 100 *μ*g/ml RNase A and propidium iodide for 15 min at 37 °C. Samples were analyzed by using flow cytometer Coulter Epics XL FACscan (Beckman Coulter, Beckman Coulter Moscow, LabTech, Russia). Cell cycle phase distribution analysis was performed with MODFIT LT 3.0 software (Verity Software House, Topsham, ME, USA).

### MTT-test for cell viability

Evaluation of cell viability was performed by using colorimetric MTT-assay (3-(4,5-Dimethylthiazol-2-yl)-2,5-diphenyltetrazolium bromide (MTT, Sigma). The amount of formazan product correlates with the quantity of live cells. Cells were seeded in 24-well plates and were treated with rapamycin or pp242. After 24 h treatment MTT (0.5 mg/ml dissolved in PBS) was added, and cells were incubated for 1.5 h at 37 °C in 5% CO_2_. The resulting formazan precipitate was dissolved in DMSO (Sigma), gently pipetted and dispensed into 96-well plate. The absorbance was measured at 570 nm wavelength using Multiskan EX (Thermo Electron, Helicon, St. Petersburg, Russia). Each experiment was repeated six times followed by calculation of the standard error of the mean.

### Immunofluorescence

Cells were seeded and grown on gelatine-coated coverslips rinsed with cold PBS, fixed in 4% formaldehyde for 15 min at RT, and permeabilized with 0.25% Triton X-100 for 20 min. After three washes cells were incubated in blocking solution (5% BSA from Sigma in PBS) and then incubated with primary antibodies against phospho-S6 (#2211 Cell Signaling Technology Europe, B.V., Leiden, The Netherlands), LC3 (#PM036 MBL, Woburn, MA, USA) at 4 °C overnight. After washing samples were incubated for 1 h with secondary antibodies AlexaFlour-488/568-conjugated goat anti-rabbit/mouse F(ab′)2 fragment (Invitrogen, Moscow, Russia). Primary and secondary antibodies were dissolved in PBS with 5% BSA and 0.1% Tween-20 (Sigma). Nuclei were stained by 5 min incubation with To-Pro3 (Invitrogen) or DAPI. Images were analyzed with the confocal microscope (Leica, Moscow, Russia).

### Protein lysates and western blotting

For immunoblotting, cell lysates were obtained by incubating cells in RIPA buffer containing PBS solution, 1% Igepal, 0.5% sodium deoxycholate, 0.1% SDS (Sigma), protease and phosphatase inhibitors (cocktail Complete, Roche, Grenzach-Wyhlen, Germany), 5 mM EGTA, 10 mM *β*-glycerophosphate). Equal amounts of protein extracts were run on polyacrylamide gel electrophoresis, transferred to PVDF-FL membranes (Millipore) and blotted with primary antibodies, according to manufacturer's recommendations. HRP-conjugated goat anti-rabbit and rabbit anti-mouse antibodies (Pierce, Thermo Fisher Science, Moscow, Russia) were used as secondary antibodies. Proteins on membranes were visualized by means of ECL (Amersham, GE Healthcare, Freiburg, Germany). The relative band intensity was quantified using the Gel-pro Analyzer software (MC Inc., Rockville, MD, USA). Pre-stained molecular weight protein standard (Sea-blue Plus 2) was used as protein marker (Novex, Life Technologies, Thermo Fisher Science, Moscow, Russia).

Primary antibody: Oct3/4 (#sc-5279) and Nanog (#sc-376915) from Santa Cruz Biotechnology (Heidelberg, Germany); *α*-tubulin (Sigma #T516 8); Sox2 (#4900), GAPDH (#2118) pULk1 Ser555 (#5869), pUlkSer757 (#6888), p-mTOR Ser2448 (#6536), pSTAT3 Tyr705 (#9145), pS6 Ser235/236 (#4858), p4EBP1 Thr37/46 (#2855), PKB/AktSer308 (#13038), PKB/AktSer473 (#4060), pERKThr202/Tyr204 (#4370) and pTSC2 Thr1462 (#3617) (Cell Signaling).

### qRT-PCR and RT-PCR

For qRT-PCR and RT-PCR total cellular RNA was isolated using TRIzol (Invitrogen) according to a manufacturer's protocol. Reverse transcription was performed with 2 *μ*g RNA, using random hexaprimers (Promega GmbH, Mannheim, Germany) and M-MuLV Revertase (RevertAid, Fermentas, Vilnius, Lithuania). qPCR was performed using the Real-Time PCR Reagent kit with SYBR Green dye and the reference dye ROX (Syntol, Moscow, Russia), on the 7500 Real-Time PCR System (Applied Biosystems, Life Technologies, Carlsbad, CA, USA). The reaction parameters were according to the manufacturer's instructions (5 min at 95 °C, then 50 s at 60 °C and 15 s at 95 °C repeated in 45 cycles).

*oct-3/4*: 5′-CAAGTTGGCGTGGAGACT-3′/5′-TTCATGTCCTGGGACTCCTC-3′ *klf-4*: 5′-GACTAACCGTTGGCGTGAG-3′/5′-CGGGTTGTTACTGCTGCAAG-3′ *nanog*: 5′-GATGCAAGAACTCTCCTCCA-3′/5′-CAATGGATGCTGGGATACTC-3′ *gapdh*: 5′-TGTGTCCGTCGTGGATCTGA-3′/5′-TTGCTGTTGAAGTCGCAGGAG-3′ *beta-actin*: 5′-CCGTAAAGACCTCTATGCCAAC-3′/5′-ATGGAGCCACCGATCCACA-3′ *fgf5:* 5′-GCTGTGTCTCAGGGGATTGT-3′/5′-CACTCTCGGCCTGTCTTTTC-3′ *T/brachyury*: 5′-TGACCAAGAACGGCAGGAGG -3′/5′-TGGGTCTCGGGAAAGCAGTG-3′ *mixL:* 5′-CGTCTTCCGACAGACCATGT-3′/5′-GTTCTGGAACCACACCTGGAT-3′ *afp:* 5′-TGGTTACACGAGGAAAGCCC-3′/5′-AATGTCGGCCATTCCCTCACG-3′ *mtor:* 5′-CAAGATGCTTGGGACGGGT-3′/5′-CATTCCGGCTCTTCAGTCCA-3′.

### Caspase-3 activity assay

Fluorescent caspase-3 substrate (Ac-DEVD-AMC, Biomol, Enzo Life Sciences via Chimmed, Moscow, Russia) was used to measure caspase-3 activity *in vitro*. Cells were washed with PBS and lysed in ice-cold buffer containing 50 mM HEPES pH 7.4, 5 mM CHAPS, 5 mM DTT, 0.5% NP-40 for 30 min. After centrifugation protein concentration was measured and 100 μg of proteins from each sample were used for the assay. Samples were incubated for 1 h at 37 °C in the following buffer: 20 mM HEPES pH 7.4, 0.1% CHAPS, 5 mM DTT, 2 mM EDTA and 40 mM Ac-DEVD-AMC (Sigma). The emission fluorescence at 460 nm produced by cleaved Ac-DEVD-AMC was measured after excitation at 360 nm using fluorimeter GloMax-Multi Jr (Promega via Dia-M, Moscow, Russia).

## Abbreviations

mESCs: mouse embryonic stem cells
LIF: Leukemia inhibitory factor
qRT-PCR: quantitative RT-PCR
MTT: (3-(4,5-Dimethylthiazol-2-yl)-2,5-diphenyltetrazolium bromide
PBS: phosphate saline solution
mTOR: Mammalian Target Of Rapamycin
EDTA: ethylenediaminetetraacetic acid
EGTA: ethylene glycol tetraacetic acid
SDS: sodium dodecyl sulfate
PVDF membrane: polyvinylidene fluoride membrane
HRP: horseradish peroxidase
ECL: enhanced chemiluminescence
HEPES: 4-(2-hydroxyethyl)-1-piperazineethanesulfonic acid
CHAPS: 3-[(3-cholamidopropyl)dimethylammonio]-1-propanesulfonate
DTT: dithiothreitol

## References

[bib1] Smith AG, Heath JK, Donaldson DD, Wong GG, Moreau J, Stahl M et al. Inhibition of pluripotential embryonic stem cell differentiation by purified polypeptides. Nature 1988; 336: 688–690. 3143917 10.1038/336688a0

[bib2] Ying Q-L, Nichols J, Chambers I, Smith A. BMP induction of Id proteins suppresses differentiation and sustains embryonic stem cell self-renewal in collaboration with STAT3. Cell 2003; 115: 281–292. 14636556 10.1016/s0092-8674(03)00847-x

[bib3] Niwa H. How is pluripotency determined and maintained? Development 2007; 134: 635–646. 17215298 10.1242/dev.02787

[bib4] Niwa H, Ogawa K, Shimosato D, Adachi K. A parallel circuit of LIF signalling pathways maintains pluripotency of mouse ES cells. Nature 2009; 460: 118–122. 19571885 10.1038/nature08113

[bib5] Tang Y, Tian XC. JAK-STAT3 and somatic cell reprogramming. JAKSTAT 2013; 2: e24935. 24470976 10.4161/jkst.24935 PMC3894236

[bib6] Nichols J, Davidson D, Taga T, Yoshida K, Chambers I, Smith A. Complementary tissue-specific expression of LIF and LIF-receptor mRNAs in early mouse embryogenesis. Mech Dev 1996; 57: 123–131. 8843390 10.1016/0925-4773(96)00531-x

[bib7] Dahéron L, Opitz SL, Zaehres H, Lensch MW, Andrews PW, Itskovitz-Eldor J et al. LIF/STAT3 signaling fails to maintain self-renewal of human embryonic stem cells. Stem Cells 2004; 22: 770–778. 15342941 10.1634/stemcells.22-5-770

[bib8] Kunath T, Saba-El-Leil MK, Almousailleakh J, Wray S, Meloche J, Smith A. FGF stimulation of the Erk1/2 signalling cascade triggers transition of pluripotent embryonic stem cells from self-renewal to lineage commitment. Development 2007; 134: 2895–2902. 17660198 10.1242/dev.02880

[bib9] Caron E, Ghosh S, Matsuoka Y, Ashton-Beaucage D, Therrien M, Lemieux S et al. A comprehensive map of the mTOR signaling network. Mol Syst Biol 2010; 6: 453. 21179025 10.1038/msb.2010.108 PMC3018167

[bib10] Gangloff YG, Mueller M, Dann SG, Svoboda P, Sticker M, Spetz J et al. Disruption of the mouse mTOR gene leads to early postimplantation lethality and prohibits embryonic stem cell development. Mol Cell Biol 2004; 24: 9508–9516. 15485918 10.1128/MCB.24.21.9508-9516.2004 PMC522282

[bib11] Murakami M, Ichisaka T, Maeda M, Oshiro N, Hara K, Edenhofer F et al. mTOR is essential for growth and proliferation in early mouse embryos and embryonic stem cells. Mol Cell Biol 2004; 24: 6710–6718. 15254238 10.1128/MCB.24.15.6710-6718.2004 PMC444840

[bib12] Zhou J, Su P, Wang L, Chen J, Zimmermann M, Genbacev O et al. mTOR supports long-term self-renewal and suppresses mesoderm and endoderm activities of human embryonic stem cells. Proc Natl Acad Sci USA 2009; 106: 7840–7845. 19416884 10.1073/pnas.0901854106 PMC2683106

[bib13] Easley CA, Ben-Yehudah A, Redinger CJ, Oliver SL, Varum ST, Eisinger VM et al. mTOR-mediated activation of p70 S6K induces differentiation of pluripotent human embryonic stem cells. Cellular Reprogram 2010; 12: 263–274. 10.1089/cell.2010.0011 PMC2993047 20698768

[bib14] Agrawal P, Reynolds J, Chew S, Lamba DA, Hughes RE. DEPTOR is a stemness factor that regulates pluripotency of embryonic stem cells. J Biol Chem 2014; 289: 31818–31826. 25258312 10.1074/jbc.M114.565838 PMC4231659

[bib15] Jiang J, Chang YS, Loh YH, Cai J, Tong GQ, Lim CA et al. A core Klf4 circuitry regulates self-renewal of embryonic stem cells. Nat Cell Biol 2008; 10: 353–360. 18264089 10.1038/ncb1698

[bib16] Adachi K, Schöler HR. Directing reprogramming to pluripotency by transcription factors. Curr Opin Genet Dev 2012; 22: 416–422. 22868173 10.1016/j.gde.2012.07.001

[bib17] Murray P, Edgar D. The regulation of embryonic stem cell differentiation by leukaemia inhibitory factor (LIF). Differentiation 2001; 68: 227–234. 11776475 10.1046/j.1432-0436.2001.680410.x

[bib18] Ying Q-L, Wray J, Nichols J, Batlle-Morera L, Doble B, Woodgett J et al. The ground state of embryonic stem cell self-renewal. Nature 2008; 453: 519–523. 18497825 10.1038/nature06968 PMC5328678

[bib19] Ma L, Chen Z, Erdjument-Bromage H, Tempst P, Pandolfi PP. Phosphorylation and functional inactivation of TSC2 by Erk implications for tuberous sclerosis and cancer pathogenesis. Cell 2005; 12: 179–193. 10.1016/j.cell.2005.02.031 15851026

[bib20] Guan J-L, Simon AK, Prescott M, Menendez JA, Liu F, Wang F et al. Autophagy in stem cells. Autophagy 2013; 9: 830–849. 23486312 10.4161/auto.24132 PMC3672294

[bib21] Jung CH, Ro S-H, Cao J, Otto NM, Kim D-H. mTOR regulation of autophagy. FEBS Lett 2010; 584: 1287–1295. 20083114 10.1016/j.febslet.2010.01.017 PMC2846630

[bib22] Kim J, Kundu M, Violet B, Guan K-L. AMPK and mTOR regulate autophagy through direct phosphorylation of Ulk1. Nat Cell Biol 2011; 13: 132–141. 21258367 10.1038/ncb2152 PMC3987946

[bib23] Hirai H, Karian P, Kikyo N. Regulation of embryonic stem cell self-renewal and pluripotency by leukaemia inhibitory factor. Biochem J 2011; 438: 11–23. 21793804 10.1042/BJ20102152 PMC3418323

[bib24] Lianguzova MS, Chuykin IA, Nordheim A, Pospelov VA. Phosphoinositide 3-kinase inhibitor LY294002 but not serum withdrawal suppresses proliferation of murine embryonic stem cells. Cell Biol Int 2007; 31: 330–337. 17321171 10.1016/j.cellbi.2007.01.019

[bib25] Coronado D, Godet M, Bourillot P-Y, Tapponnier Y, Bernat A, Petit M et al. A short G1 phase is an intrinsic determinant of naïve embryonic stem cell pluripotency. Stem Cell Res 2013; 10: 118–131. 23178806 10.1016/j.scr.2012.10.004

[bib26] Santostefano KE, Hamazaki T, Pardo CE, Kladde MP, Terada N. Fibroblast growth factor receptor 2 homodimerization rapidly reduces transcription of the pluripotency gene *Nanog* without dissociation of activating transcription factors. J Biol Chem 2012; 287: 30507–30517. 22787153 10.1074/jbc.M112.388181 PMC3436299

[bib27] Copp J, Manning G, Hunter T. TORC-specific phosphorylation of mTOR: phospho-Ser2481 is a marker for intact mTORC2. Cancer Res 2009; 69: 1821–1827. 19244117 10.1158/0008-5472.CAN-08-3014 PMC2652681

[bib28] Wu Y, Li Y, Zhang H, Huang Y, Zhao P, Tang Y et al. Autophagy and mTORC1 regulate the stochastic phase of somatic cell reprogramming. Nat Cell Biol 2015; 17: 715–725. 25985393 10.1038/ncb3172

[bib29] Wang S, Xia P, Ye B, Huang G, Liu J, Fan Z. Transient activation of autophagy via Sox2-mediated suppression of mTOR is an important early step in reprogramming to pluripotency. Cell Stem Cell 2013; 13: 617–625. 24209762 10.1016/j.stem.2013.10.005

[bib30] Nuschke A, Rodrigues M, Stolz D, Chu CT, Griffith L, Wells A. Human mesenchymal stem cells/multipotent stromal cells consume accumulated autophagosomes early in differentiation. Stem Cell Res Ther 2014; 5: 140. 25523618 10.1186/scrt530 PMC4446103

[bib31] Cho YH, Han KM, Kim D, Lee J, Lee SH, Choi KW et al. Autophagy regulates homeostasis of pluripotency-associated proteins in hESCs. Stem Cells 2014; 32: 424–435. 24170349 10.1002/stem.1589

[bib32] Mizushima N, Yoshimori T, Levine B. Methods in mammalian autophagy research. Cell 2010; 140: 313–326. 20144757 10.1016/j.cell.2010.01.028 PMC2852113

[bib33] Nichols J, Smith А. Naive and primed pluripotent states. Cell Stem Cell 2009; 4: 487–492. 19497275 10.1016/j.stem.2009.05.015

[bib34] Guo G, Yang J, Nichols J, Hall JS, Eyres I, Mansfield W et al. Klf4 reverts developmentally programmed restriction of ground state pluripotency. Development 2009; 136: 1063–1069. 19224983 10.1242/dev.030957 PMC2685927

[bib35] Yang J, van Oosten AL, Theunissen TW, Guo G, Silva GC, Smith A. Activation of LIF/JAK/STAT3 is a limiting component for the induction of naive pluripotency Stat3 activation is limiting for reprogramming to ground state pluripotency. Cell Stem Cell 2010; 7: 319–328. 20804969 10.1016/j.stem.2010.06.022 PMC3459098

[bib36] Brons IG, Smithers LE, Trotter MW, Rugg-Gunn P, Sun B, Chuva de Sousa Lopes SM et al. Derivation of pluripotent epiblast stem cells from mammalian embryos. Nature 2007; 448: 191–195. 17597762 10.1038/nature05950

[bib37] Tesar PJ, Chenoweth JG, Brook FA, Davies TJ, Evans EP, Mack DL et al. New cell lines from mouse epiblast share defining features with human embryonic stem cells. Nature 2007; 448: 196–199. 17597760 10.1038/nature05972

[bib38] Rossant J. Stem cells and early lineage development. Cell 2008; 132: 527–531. 18295568 10.1016/j.cell.2008.01.039

[bib39] Chen X, Xu H, Yuan P, Fang F, Huss M, Vega VB et al. Integration of external signaling pathways with the core transcriptional network in embryonic stem cells. Cell 2008; 133: 1106–1117. 18555785 10.1016/j.cell.2008.04.043

[bib40] van Oosten AL, Costa Y, Smith A, Silva JC. JAK/STAT3 signalling is sufficient and dominant over antagonistic cues for the establishment of naive pluripotency. Nat Commun 2012; 3: 817. 22569365 10.1038/ncomms1822 PMC3567838

