# Supplementary material for: Leukemia inhibitory factor (LIF) withdrawal activates mTOR signaling pathway in mouse embryonic stem cells through the MEK/ERK/TSC2 pathway
Source: Cell Death Dis. 2016 Jan 14;7(1):e2050–. doi: 10.1038/cddis.2015.387 (PMC4816172; doi:10.1038/cddis.2015.387)
Supplement: Supplementary Figure S1 [file cddis2015387x1.ppt]

## Slide 1
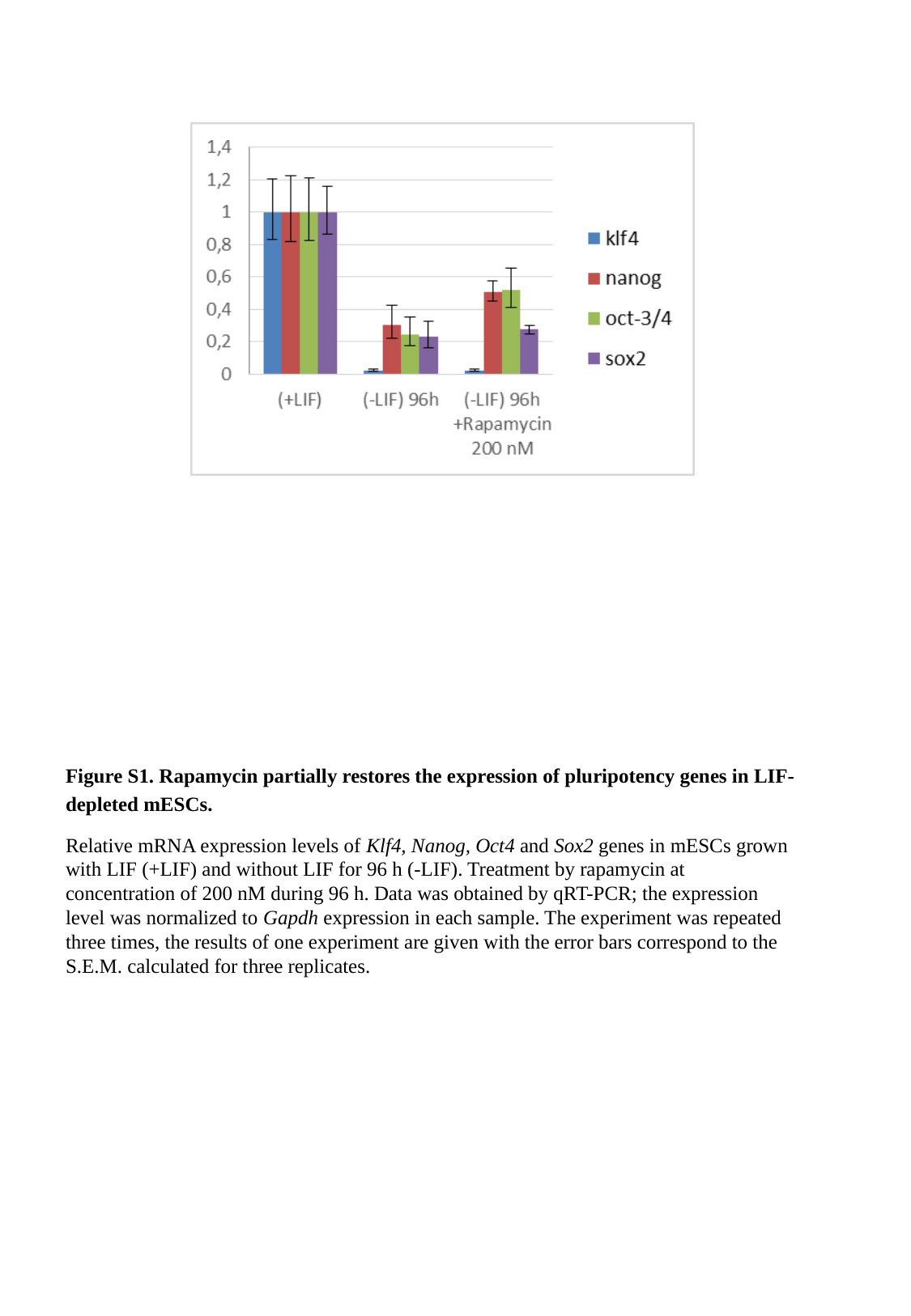

Figure S1. Rapamycin partially restores the expression of pluripotency genes in LIF-depleted mESCs.
Relative mRNA expression levels of Klf4, Nanog, Oct4 and Sox2 genes in mESCs grown with LIF (+LIF) and without LIF for 96 h (-LIF). Treatment by rapamycin at concentration of 200 nM during 96 h. Data was obtained by qRT-PCR; the expression level was normalized to Gapdh expression in each sample. The experiment was repeated three times, the results of one experiment are given with the error bars correspond to the S.E.M. calculated for three replicates.
